# Intravenous versus subcutaneous route pharmacokinetics of paracetamol (acetaminophen) in palliative care patients: study protocol for a randomized trial (ParaSCIVPallia)

**DOI:** 10.1186/s13063-019-3969-0

**Published:** 2020-02-04

**Authors:** Marine Vernant, Marie Lepoupet, Christian Creveuil, Antoine Alix, Charlotte Gourio, Laure Peyro-Saint-Paul, Veronique Lelong-Boulouard, Cyril Guillaumé

**Affiliations:** 1 Pain and Palliative Care Department and Regional Palliative Care Unit (RPCU) Maurice Abiven of Fondation de la Miséricorde, Caen University Hospital, CHU de Caen- avenue de la côte de Nacre, 14000 Caen, France; 2 Pain and Palliative Care Department, Caen University Hospital, 14000 Caen, France; 3 Caen University Hospital, Pharmacy, 14000 Caen, France; 4 Clinical Research Department, Caen University Hospital, 14000 Caen, France

**Keywords:** Paracetamol, Acetaminophen, Subcutaneous route, Intravenous route, Pharmacokinetics, Palliative care

## Abstract

**Background:**

Among palliative care (PC) patients who are administered paracetamol, the subcutaneous (SC) route is often an alternative to the intravenous (IV) route. Yet pharmacological and clinical data on whether these are equivalent pharmacokinetically are lacking. Many French palliative teams are now empirically using paracetamol by the SC route, but there are no data to support this practice. This trial aims to compare the pharmacokinetic (PK) parameters of paracetomol between the IV and SC routes in PC patients.

**Methods/design:**

This is a randomized, open, crossover study in two PC centers. The primary endpoints are AUC0-t, AUC0-∞, Cmax, Vd, and t1/2. All adverse events will be reported for a safety analysis. Twenty adult PC patients with an IV device having spontaneous pain not related to care, with a numeric pain rate scale > 3/10, or having a systematic prescription of paracetamol as the usual treatment will be included. All patients also have to meet all eligibility criteria.

**Conclusion:**

This is the first study comparing PK parameters for IV paracetamol versus SC paracetamol in PC patients.

**Trial registration:**

ClinicalTrials.gov, NCT03944044. Registered on 4 June 2019.

Committee for the protection of persons (CPP) 18.09.05.58206 approval 4 October 2018.

National Drug Safety Agency (ANSM; Agence Nationale de Sécurité Médicament) MEDAECNAT-2018-09-00009 approval 29 November 2018.

## Background

In France, paracetamol (acetaminophen) is currently the first-line non-opioid treatment used in pain management. This treatment is administered by different routes depending on the patient’s circumstances.

In palliative care (PC), many injectable drugs are administered by the subcutaneous (SC) route [1, 2] to prevent patients from developing a venous track and receiving iterative punctures or for patients with damaged venous access. Many French palliative teams are now empirically using paracetamol by the SC route [3]. A recent French study carried out by Leheup et al. [3], conducted in 160 patients in the PC units of three hospitals in France from 2014 to 2017, evaluated the tolerability of SC paracetamol administration in a prospective multicenter observational study. Of the 160 patients, 44 (28%) experienced at least one non-serious local adverse event (edema in 29, erythema in 5, pain in 15, hematoma in 2, pruritus in 1, and localized inflammation in 2). No serious adverse events were observed. Factors associated with the occurrence of local adverse events were younger age, administration in the arm and thorax, and a high number of daily administrations. At minimum, this study shows good safety of paracetamol using the SC route.

Sometimes no other drug or route of administration is possible, for example, if venous access is excessively poor, oral intake is not possibile, or the patient cannot be mobilized to use the rectal route of administration. Furthermore, the rectal route has variable bioavailability.

We therefore designed a study to compare the pharmacokinetics (PK) of SC and intravenous (IV) routes in the same patient in a PC situation to determine if the PK are equivalent between these two modes of administration.

If the IV and SC routes are similar, a larger study should be done on a population of PC patients to evaluate safety and efficacy of the SC route for paracetamol administration.

## Methods/design

The design of this study is based on other PK studies [4–8].

### Study setting

This is a comparative, randomized, open, crossover, bicenter clinical trial (Pain and PC Department of University Hospital, Caen, France and Regional PC Unit, Fondation de la Miséricorde, Caen, France) with SC and IV paracetamol injections successively given to each patient.

This study received ethics approval by the Committee for the Protection of Persons (CPP) and National Drug Safety Agency. All investigators have received “Good Clinical Practice” training and the sponsor monitored the study according to these recommendations. All the quantitative analyses will be conducted according to the principles of Good Laboratory Practice.

### Objectives

The main objective is to demonstrate a PK equivalence between the two routes of administration (IV and SC). The secondary objectives are to compare the efficacy of these two modes of administration with regards to pain management and to explore global and cutaneous tolerance of paracetamol administered by the SC route.

### Outcomes

The main measurement will be paracetamol blood concentration.

Curves will be established for each patient, and we will determine and compare for each route of administration and for each patient AUC0-t, AUC0-∞, Cmax, Vd, and t1/2. Data will also permit us to compare PK characteristics between all of the patients.

Secondary evaluation criteria will be pain evaluation by Numeric Pain Rate Scale (NPRS) throughout the duration of the protocol and clinical evaluation of safety by the nurse.

### Eligibility criteria

#### Inclusion criteria

1. Patients ≥ 18 years old, hospitalized
2. Patients in PC
3. Patients having an IV device with the presence of venous reflux (implantable venous site, PICC line, central track)
4. Patient who have a prescription for Paracetamol four time a day even if there is no pain evaluation
5. Patients able to self-evaluate their pain by NPRS
6. No contraindications to paracetamol
7. No contraindications to alternative analgesics (low and strong opioids, non-steroidal anti-inflammatory drugs)
8. Possibility to not take paracetamol in the previous 24 h before inclusion
9. Patients with a blood test dating back less than 7 days, without severe renal (DFG > 30) or hepatic failure (glutamic pyruvic transaminase (SGPT)/serum glutamic-oxaloacetic transaminase (SGOT) > 350 UI/L, bilirubin > 40 μmol/L, TP < 50%)
10. Patients in a French social security regime
11. Patients who agree to participate in the study with written consent

#### Exclusion criteria

1. Patients under legal protection
2. Patients who participate in another study less than 30 days before
3. Patients weighing less than 50 kg
4. Patients having a contraindication to the SC route
5. Pregnant or breastfeeding woman
6. Patients administered paracetamol less than 24 h before the beginning of the inclusion
7. Patients administered a weak opioid less than 2 h before or a strong opioid less than 1 h before the beginning of administration of paracetamol
8. Patients having a fever
9. Patients unable to communicate

### Experimental plan/intervention

#### Sample size

As we could not find any information in the literature on the variability of PK parameters in a palliative care population, we relied on the number of subjects included in clinical studies with similar methodologies [4, 9–12] and decided to include 20 patients. This number is also in line with the recommendations of the European Medicines Agency [13], which sets at 12 the minimum number of patients to be included in a bioequivalence trial.

Patients who do not complete the study will be replaced by new patients in order to reach a total of 20 subjects in the statistical analysis.

#### Protocol steps

**Fig. 1:**
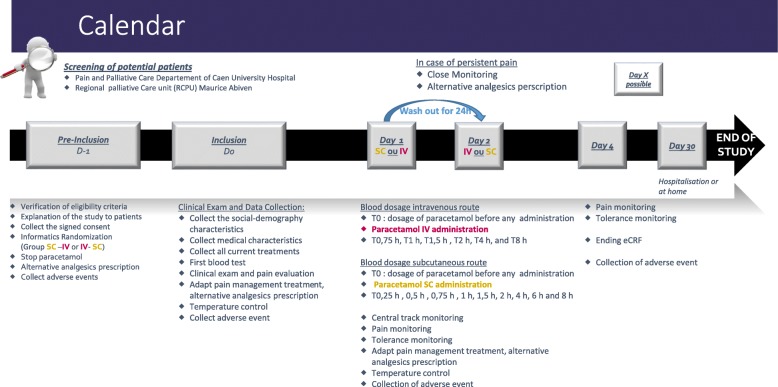
Protocol design

The study design is described in Fig. 1.

**Fig. 2:**
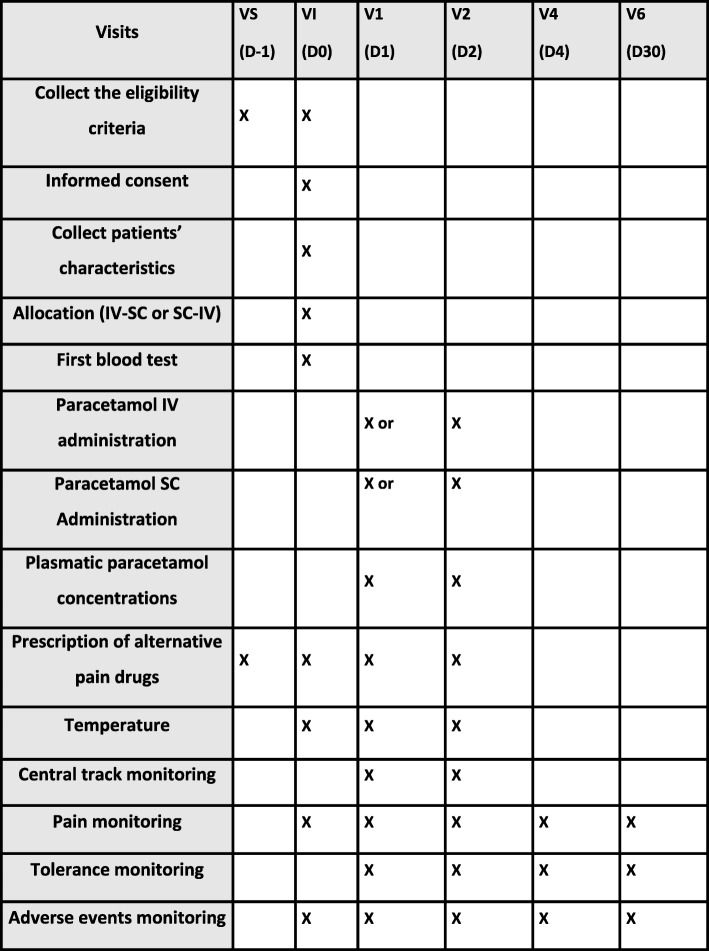
Protocol steps and intervention: SPIRIT figure

All the study was design in the respect of the SPIRIT and CONSORT advice of Additional files 1 and 2. All steps are outlined in Fig. 2 with the SPIRIT advice.

##### Pre-inclusion

Patients will be screened by investigators according to eligibility criteria. Patients will sign a consent form after receiving oral and written information from a physician investigator involved in this project.

After inclusion, they will be randomly assigned using block randomization (using Ennov clinical® software based on a list prepared by the study statistician) to SC before IV paracetamol injection or IV before SC with a washout period of 24 h between the two injections. This washout period was determined since the half-life of paracetamol has been commonly reported (Summary of product characteristics) as ranging from 1 to 4 h depending on the administration route. Almost complete elimination may be considered after five to six half-lives; thus, after 24 h, paracetamol can be considered completely eliminated.

##### Inclusion

Day −1:

- Verification of eligibility criteria
- Explanation of the study to patients: information about predictable modalities, constraints, and risks of the study
- Collect signed consent
- Block randomization (SC–IV or IV—SC group)
- Stop paracetamol
- Alternative analgesic prescription

Day 0:

- Collect patient characteristics: temperature, size, gender, liver and kidney history, main disease. The specific body weight to be used for drug dosing and pharmacokinetic calculations is also collected.
- Collect information on all current treatments.
- Adapt pain management treatment and if paracetamol is included in the patient’s treatment regimen, the practitioner must prescribe alternative analgesics.
- Plan for collection of information on alternative analgesics from day 1 to day 2: hours of administration and dosage.
- First blood test: albumin, liver function tests (SGOT, SGPT, gamma-glutamyltransferase, alkaline phosphatase), kidney function tests (creatinine, glomerular filtration rate), human chorionic-gonadotropin, beta subunit) (only childbearing women).

Day 1:

- Determine concentration of paracetamol in blood before any administration (TO); the blood sample must be taken at the precise time specified in the protocol.
- Administration of 1000 mg of paracetamol by the first route of administration designated by randomization using infusion pump Volumat® or Volumed® at a rate of 100 ml in 30 min (200 mL/h).
- Carry out blood quantitative analysis of paracetamol according to the following parameters and depending on the first route of administration:
- IV route: after paracetamol administration and at every blood draw, rinse the central track using 10 mL of physiological serum; blood taken at 0.75, 1, 1.5, 2, 4, and 8 h
- SC route: after every blood draw, rinse the central track using 10 mL of physiological serum; blood taken at 0.25, 0.5, 0.75, 1, 1.5, 2, 4, 6, and 8 h
- At each blood test, the patient will undergo a NPRS and an assessment of the tolerance.

Day 2:

Twenty-four hours after first administration of paracetamol (washout), patients will receive paracetamol by the second route of administration according to the randomization and the same timeline as on day 1.

At any point pain is not well controlled, the investigator practitioner must prescribe alternative drugs and treatment considered necessary for the patient to obtain relief. Any adverse events, other unidentified effects of trial intervention, and any alternative drugs used have to be listed in the electronic case report form (eCRF) of the patient and declared to the promoter, following the procedure described in the protocol.

##### End of protocol visit

Every patient will have an exam on the fourth and 30th days of the protocol for safety monitoring of the SC route of administration. In case of adverse events, long-term monitoring will be proposed and a specialist opinion will be requested if the investigating physician or the promoter deems it necessary.

### Data collection methods

Due to the study methodology, patients and investigators cannot be blinded. The outcome assessors will not be blinded either.

Every participant will be assigned a unique eCRF.

#### Pharmacokinetic data

##### Pharmacokinetic sampling procedure

Blood samples (2 ml) will be collected in dry tubes free of gel at 0.75, 1, 1.5, 2, 4, and 8 h after paracetamol IV administration or 0.25, 0.5, 0.75, 1, 1.5, 2, 4, 6, and 8 h after SC administration. The samples will be rapidly transported to the laboratory.

##### Assay method

After centrifugation, serum samples will be immediately used for quantification of acetaminophen with the EMIT tox TM Acetaminophen Assay, which is a homogeneous enzyme immunoassay performed in AU 5800 clinical chemistry systems (Beckman Coulter, France). The limit of quantification is 0.12 mg/L. Precision is better than 6% for three quality control levels. The quantitative analyses will be conducted according to the principles of Good Laboratory Practice.

##### PK calculations

Paracetamol concentrations obtained after IV and SC administration will be analyzed using a one-compartment open model (because paracetamol PK is known to be linear). The apparent first order elimination rate constant (ke) and the corresponding apparent elimination half-life (t1/2 = Ln2/ke) will be determined by least squares regression analysis of the terminal phase of the serum concentration–time curve. The apparent first order absorption rate (ka) and the corresponding apparent resorption half-life (t1/2 = Ln2/ka) will be determined by least squares regression analysis of the resorption phase from the serum concentration–time curve obtained after SC administration. The maximum observed serum concentration (Cmax) and the time required to reach Cmax (Tmax) will be calculated using the following formula: Tmax = Ln(Ka/Ke)/(Ka − Ke) and Cmax = C0 × (e^−Ke^ × Tmax − e^−Ka^ × Tmax). AUC0-∞ will be calculated using the linear trapezoidal rule over the interval of 0 to 8 h (AUC0–8 h) and extrapolated to infinity with the following equation: AUC0-∞ = AUC0–8 h + C8 h/ke. The bioavailability (f) corresponds to the ratio AUC0-∞ (after SC administration)/AUC0-∞ (after IV administration). The volume of distribution will be evaluated with the equations Vd = D/C0 (IV) and Vd = f × D/C0 (SC). The total clearance (CL) will be calculated using the equation CL = Ke × Vd.

#### Data analysis

The PK parameters AUC0-t, AUC0-∞, Cmax, Vd, and t_1/2_, whose distributions are known to be approximately log-normal, will be described for the two modes of administration in the form of geometric means with geometric coefficients of variation. Tmax will be summarized by a median and quartiles.

The parameters AUC0-t, AUC0-∞, Cmax, Vd, and t_1/2_ will then be log-transformed and compared between the two modes of administration using a linear regression model taking into account the following effects: treatment sequence (IV–SC or SC–IV), subjects (nested in the treatment sequence), treatment period (period 1 or period 2), and mode of administration (IV or SC). For each parameter, the difference between the two modes of administration will be tested based on the *P* value associated with the treatment effect. A non-parametric test (Wilcoxon signed-rank test) will be used for Tmax.

The bioequivalence between the two modes of administration will be tested for AUC0-t, AUC0-∞, and Cmax based on the 90% confidence interval of the ratio of the geometric means (SC vs IV). According to the recommendations of the European Medicines Agency [13], we can conclude bioequivalence if the confidence interval is fully within the range 80–125%.

Pain scores will be compared between the two modes of administration by paired *t*-tests or Wilcoxon signed-rank tests, depending on the form of the distribution.

Data will be analyzed based on a per protocol analysis. Statistical significance will be set at *P* < 0.05. Data will be analyzed at the Biostatistics and Clinical Research Unit of Caen University Hospital, with SPSS and R software.

#### Adverse events data

Information on all adverse events will be collected, from consent to the end of patient participation, and reported to the sponsor. Serious adverse reactions (SAR) will be qualified as expected or unexpected by the sponsor. Expected SAR related to paracetamol are thrombocytopenia, leucopenia, neutropenia, hypersensitivity, hypotension, increased transaminases, malaise, tachycardia, flushing, pruritus, and erythema. Other SAR will be considered as unexpected.

A Data Safety Monitoring Board (DSMB) will review the safety independently. The DSMB will include three experts: one pharmacologist, one physician pain specialist, and one dermatologist. The sponsor will request DSMB advice in case of suspected unexpected SAR or any new safety information. The DSMB is charged with providing advice to the sponsor recommendations that include (a) continuation of the study, (b) continuation with modification, and (c) termination of the study.

#### Potentially interacting drugs

In view of the PC setting and the diversity of treatments received by patients, we chose to collect through the eCRF information on every treatment taken by the patient during the study.

Some of these treatments could alter the SC PK parameters and will be studied more closely and discussed in cases of strong differences between patients. This was not one of our objectives and will need a further study. We know that administration of probenecide or salycilamide, for example, could alter the IV PK of paracetamol, but this does not presume any impact on SC PK. Furthermore, because each patient will be administered paracetamol by both routes, we will be able to discuss this potential interaction.

## Discussion

Many drugs could be administered by the SC route in PC [1, 2]. For analgesic drugs, strong and weak opioids are currently used by the SC route, with an equivalence dose from the IV route calculated according to well-known conversion factors [14].

However, there is no study highlighting the equivalence, efficacy, and safety of paracetamol administered subcutaneously. Currently, increasingly more PC teams empirically choose to use this method of administration for patients where administration via the venous or oral routes is not possible. PC practitioners need more evidence-based data, particularly for their frail patients. In these situations, it is difficult to extrapolate from studies in other populations.

Our PC study evaluates the pharmacokinetic equivalence between SC and IV routes. It could help to determine whether the SC route is a good alternative method of administration for paracetamol.

Our study is the first pharmacokinetic analysis of subcutaneous administration of paracetamol and is one of the first pharmacokinetic studies on PC patients. It is a real opportunity to show that studies are technically possible in the PC setting.

### Limitations

There are several limitations to our study.

Firstly, our study was not designed to investigate clinical outcomes of efficacy or safety of SC paracetamol. This is a pharmacokinetic analysis, comparing SC and IV PK. The practical implications of our results need to be confirmed by a larger, dedicated trial. The efficacy should be studied on a larger sample in order to compare pain before and after the injection. With regards to safety, a larger sample is also needed to conclude whether SC administration has good tolerance.

Secondly, intra- and inter-individual variation in pharmacokinetics will not be statistically analyzed in our study. We know that this could have an impact on our results but the study was not designed to answer this question. We wanted first to search for bioequivalence between IV and SC paracetamol blood concentration and PK parameters.

Thirdly, the study location at two centers will help to find patients that meet the inclusion criteria but it could change the way data are collect and introduce limitations. However, the quantitative analysis will be conducted by the same laboratory.

Finally, performing a pharmacokinetic analysis on a PC population creates additional challenges. It could be difficult to obtain consent from these frail patients. Nevertheless, the implementation of this study is a first step for all PC researchers and practitioners to develop even more PC studies.

## Conclusions

The ethics committee/institutional approval show that pharmacokinetic clinical trials can be carried out for PC patients.

If an equivalence between the SC and IV routes for PC patients is demonstrated, paracetamol may be used by PC teams in a more consensual, secure, and scientifically proven way. However, more studies will be necessary to confirm the clinical efficacy.

## Trial status

Protocol version number 3: 04/12/2018. Recruitment began in April 2019 and will probably be completed in April 2021.

## Supplementary information

**Additional file 1.** SPIRIT 2013 checklist: Recommended items to address in a clinical trial protocol and related documents.

**Additional file 2.** CONSORT 2010 checklist of information to include when reporting a randomized trial.

## Data Availability

Anonymized data can be made available to investigators upon request to the corresponding author.

## Abbreviations

GFR: Glomerular Filtration Rate
IV: Intravenous
NPRS: Numeric Pain Rate Scale
PC: Palliative care
PK: Pharmacokinetic
RPCU: Regional Palliative Care Unit
SC: Subcutaneous
SGOT: Serum glutamic-oxaloacetic transaminase
SGPT: Serum glutamic pyruvic transaminase
TP: Prothrombin Time
βhCG: Human chorionic-gonadotropin, beta subunit
